# High yield matrix-free ionization of biomolecules by pulse-heating ion source

**DOI:** 10.1038/s41598-017-15259-y

**Published:** 2017-11-09

**Authors:** Xi Luo, Phan-Trong Tue, Kiyotaka Sugiyama, Yuzuru Takamura

**Affiliations:** School of Materials Science, Japan Advanced Institute of Science and Technology (JAIST), 1-1 Asahidai, Nomi, Ishikawa, 923-1211 Japan

## Abstract

Matrix-assisted laser desorption/ionization (MALDI) mass spectrometry has been widely used for biomolecular analysis. However, with conventional MALDI, it is difficult to analyse low-molecular-weight compounds because of the interference of matrix ion signals. Here, we report a matrix-free on-chip pulse-heating desorption/ionization (PHDI) method for a wide range of biomolecules ranging from low molecular-weight substances such as glycine (75.7 Da) to large species such as α-lactalbumin (14.2 kDa). Compared with the conventional MALDI, the matrix-free PHDI method affords high yields of singly charged ions with very less fragmentation and background using only one-pulse without light (laser). We believe that this new technique for matrix-free biomolecules analysis would overcome the limitations of the conventional MALDI.

## Introduction

Matrix-assisted laser desorption/ionization mass spectrometry (MALDI-MS) has been widely used for biomolecule analysis in the last thirty years^1,2^. MALDI provides singly charged ions, resulting in simple and easy-to-interpret mass spectra. A matrix, which is added for co-crystallization with the analyte, is essentially utilized for absorbing the laser energy in MALDI. However, the use of a matrix may lead to several problems. First, the analysis of low-molecular-weight samples is frequently hindered by serious matrix-signal interference in the low mass region^3^. Second, finding a suitable matrix for the target sample often involves a trial-and-error process^4^. Third, the “sweet spot” formed by the heterogeneous co-crystallization of the matrix and the analyte may affect the intensity and reproducibility of the ion signals^5^. Besides, the ionization efficiency of neutral molecules such as carbohydrates is relatively low since they lack acidic or basic sites for receiving charges from the matrix^6^. To solve these problems, inorganic matrices, such as metallic micro- or nano- particles^7–9^, and modified Fe_3_O_4_ magnetic nanoparticles^10^, have been developed. A surface-assisted laser desorption/ionization (SALDI) method also has been developed for avoiding these matrix effects^3,11^. In SALDI, a nanostructure material is used to modify the surface of the substrates and replace the function of the matrix^12–14^. Nevertheless, the use of nanostructured materials raises several issues, such as the decrease in ionization efficiency due to surface oxidation^15^, contamination introduced by nanoparticles^16^, and signal intensity loss due to the attenuation of the laser signal in the porous substrate^13^. In addition, the preparation process used for the nanostructured materials also affects to the MS performance^17,18^. Because of such limitations, this two methods have not been very successful so far, and challenges still remain in the matrix-free analysis of biomolecules.

Recently, we have reported an on-chip pulse-heating desorption/ionization (PHDI) source for a miniaturized MS system^19^. With the PHDI method, pulsed Joule heating is used for sample desorption and ionization. An excellent performance of protein desorption/ionization was demonstrated with matrix. Singly charged and multiple-charged protein ion signals were obtained successfully under thermal energy, without light (laser). However, the fragmentation ion signals and matrix signals were also observed in the low mass region.

In this paper, an oxygen plasma treatment was introduced for chip pre-treatment to increase the wettability of the micro-heater surface, which led to significant improvements in the sample film quality (thickness and uniformity). With such a uniform and thin sample film, only singly-charged ion signal was obtained with very less background and fragmentation ion signals. Further, the protein sample could be desorbed and ionized without the matrix. This feature is well suited for the analysis of small biomolecules such as oligosaccharides, which are difficult to be directly ionized by the conventional MALDI^7,20^. The matrix-free PHDI method was also demonstrated with a wide range of biomolecules, including amino acids, peptide, and proteins. These mass spectra were obtained by only one pulse-heating shot without accumulation, using a very small amount of the sample, suggesting that the PHDI affords a high yield.

## Results

### Matrix-assisted pulse-heating desorption/ionization

**Figure 1:**
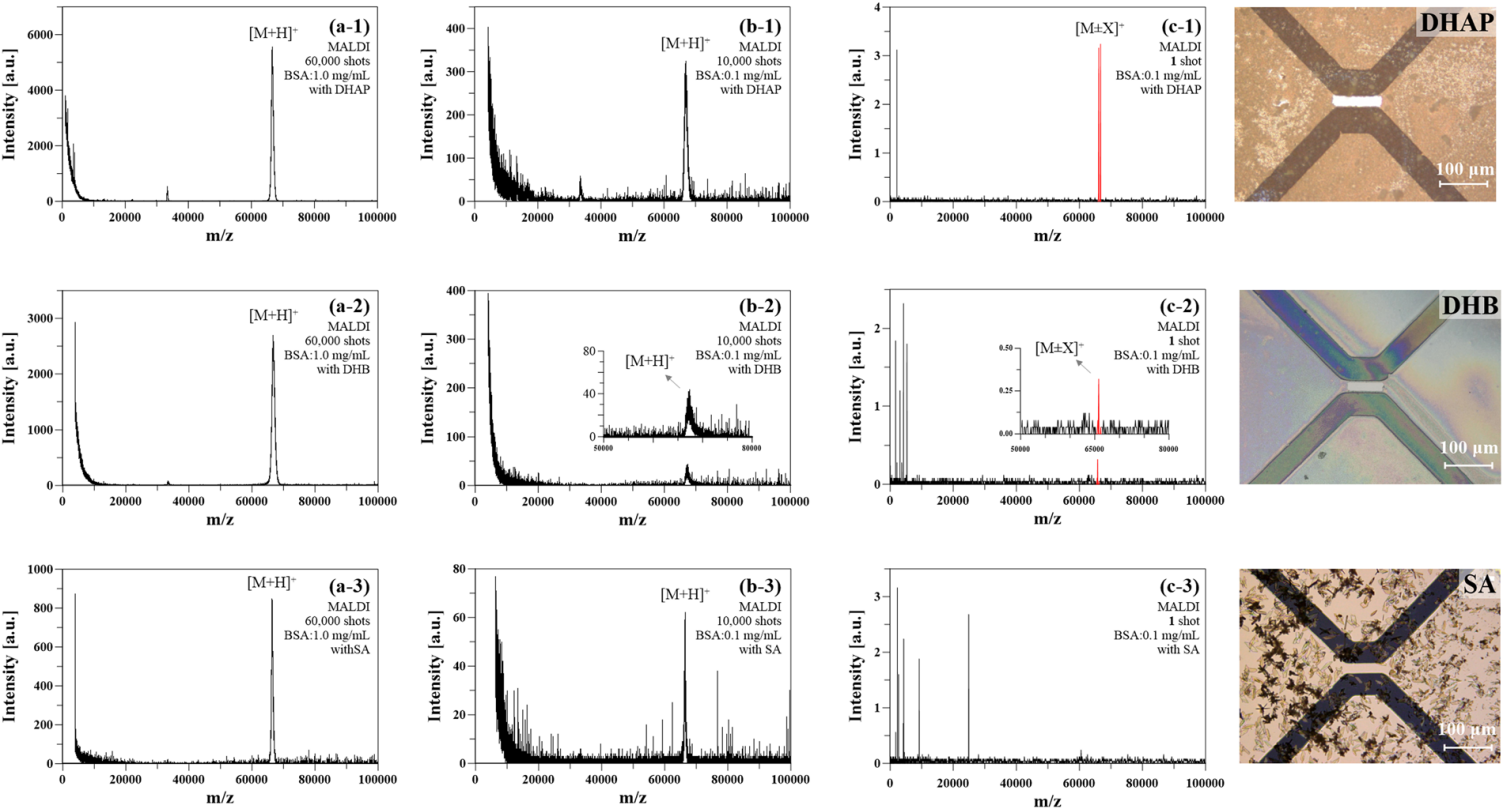
Comparison of conventional MALDI and PHDI method. (**a**) MALDI mass spectra obtained from BSA (1.0 mg/mL) mixed with DHAP (a-1), DHB (a-2), and SA (a-3) by 60,000 shots averages. (**b**) MALDI mass spectra obtained from BSA (0.1 mg/mL) mixed with DHAP (b-1), DHB (b-2), and SA (b-3) by 10,000 shots averages. (**c**) PHDI mass spectra obtained by BSA (0.1 mg/mL) mixed with DHAP (b-1), DHB (b-2), and SA (b-3) by 1 shot. Inset figures show the microscopic image of the PHDI source. After one pulse-heating shot, the sample at the centre (ionization zone) was desorbed and ionized.

For the initial investigation, bovine serum albumin (BSA) and cytochrome c (Cyt c) were used as protein samples and subjected to analysis with the matrix by the PHDI method; the mass spectra were compared with the results from MALDI. Three commonly used MALDI matrices were tested, including 2,5-dihydroxyacetophenone (DHAP), 2,5-dihydroxybenzoic acid (DHB), and sinapic acid (SA)^21,22^. For protein analysis, 1.20 × 10^−2^ μJ/μm^2^ of pulse-heating energy was supplied. As shown in Fig. 1 for BSA and SI-Fig. 1 (in Supplementary information) for Cyt c, the singly charged ion signals could be obtained by both methods with a suitable matrix. It is important to mention that only one pulse-heating shot was applied for the PHDI (Fig. 1c), while over 10,000 times shots were utilized for MALDI (Fig. 1b and SI-Fig. 1a). With the same concentration of the BSA sample solution, a low-intensity [M + H]^+^ signal was observed by MALDI (Fig. 1b), while a relatively high signal-to-noise ratio (S/N) signal was obtained by PHDI with DHAP as the matrix (Fig. 1c-1). From the S/N value, the limit of detection was estimated to be 0.01 mg/mL for Fig. 1b-1, and 0.003 mg/mL for Fig. 1c-1. Increasing the number of laser shots or the sample concentration is another way for increasing the signal intensity (Fig. 1a). Besides, strong background noise was observed in the MALDI method, especially in the low mass region. In this paper, the [M ± X]^+^ is used to represent the singly charged ion since the ion species generated by the PHDI method are still unclear.

### Matrix-free protein desorption/ionization

**Figure 2:**
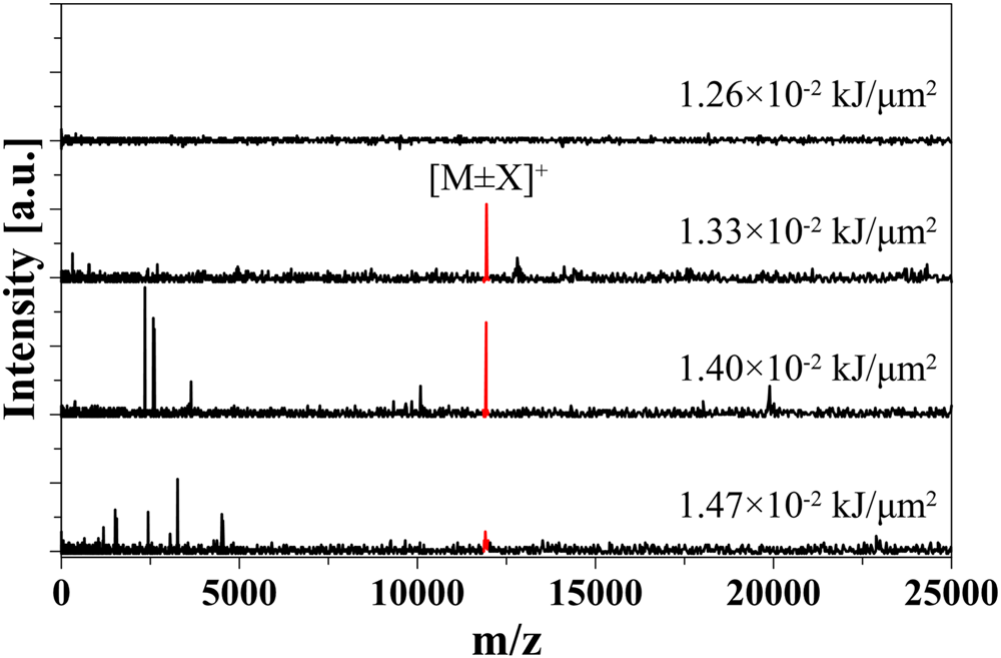
Matrix-free Cyt c (1 mg/mL) analysis by PHDI with different levels of pulse-heating energy.

**Figure 3:**
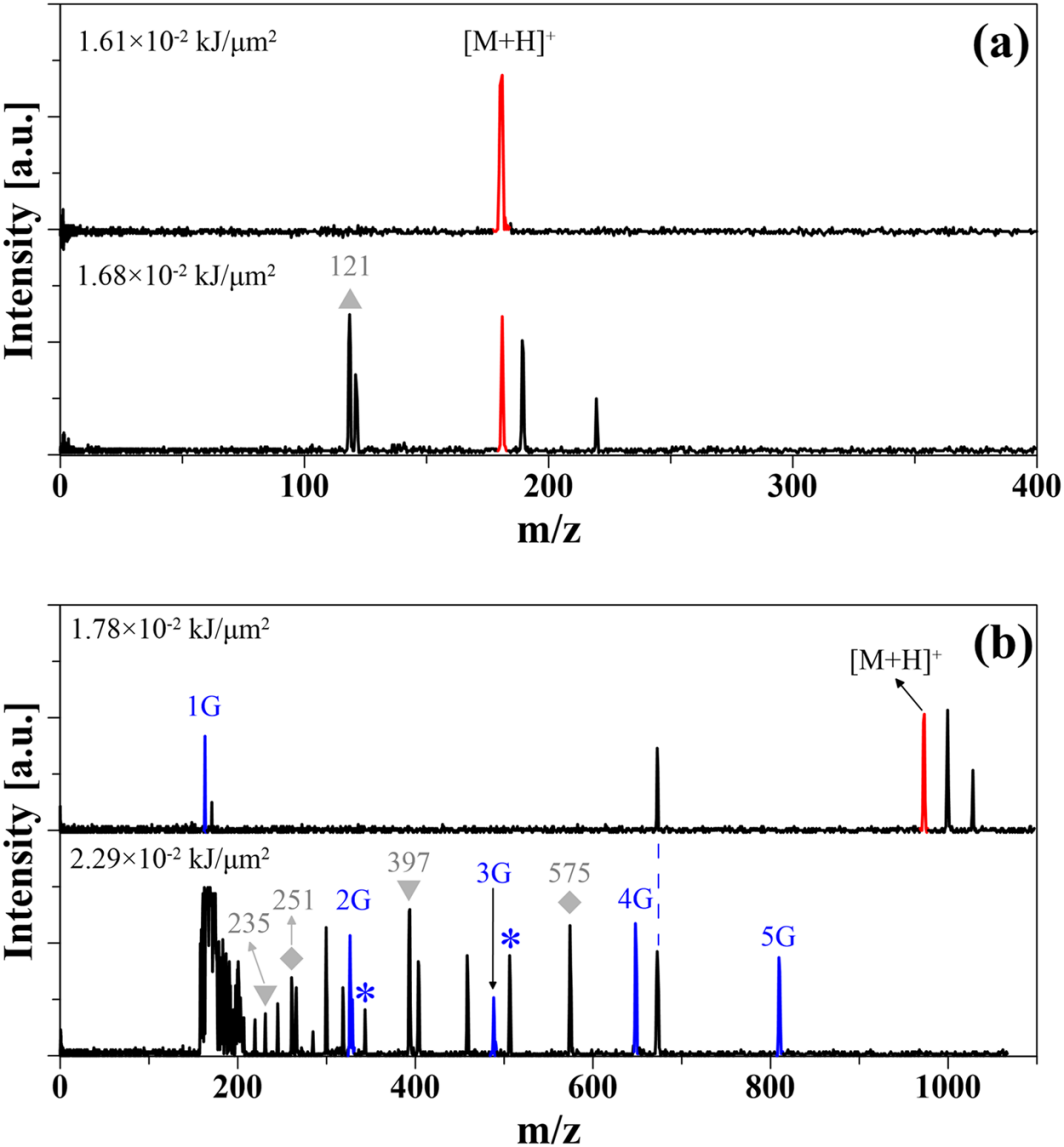
Matrix-free carbohydrates analysis by the PHDI with two levels of energy supplied. (**a**) Glucose (1 mg/mL); (**b**) α-CD (1 mg/mL). G = glucose units. Fragmentation ion signals marked with an asterisk are possibly derived from [2 G + H_2_O]^+^ and [3 G + H_2_O]^+^. Fragmentation ion signals marked with ▲ ([C_4_H_9_O_4_]^+^), ◆ ([2G-C_3_H_5_O_3_]^+^, [3G-C_3_H_5_O_3_]^+^) and ▼ ([2G-C_3_H_5_O_2_]^+^, [4G-C_3_H_5_O_2_]^+^) may due to cross-ring cleavage^25^.

**Figure 4:**
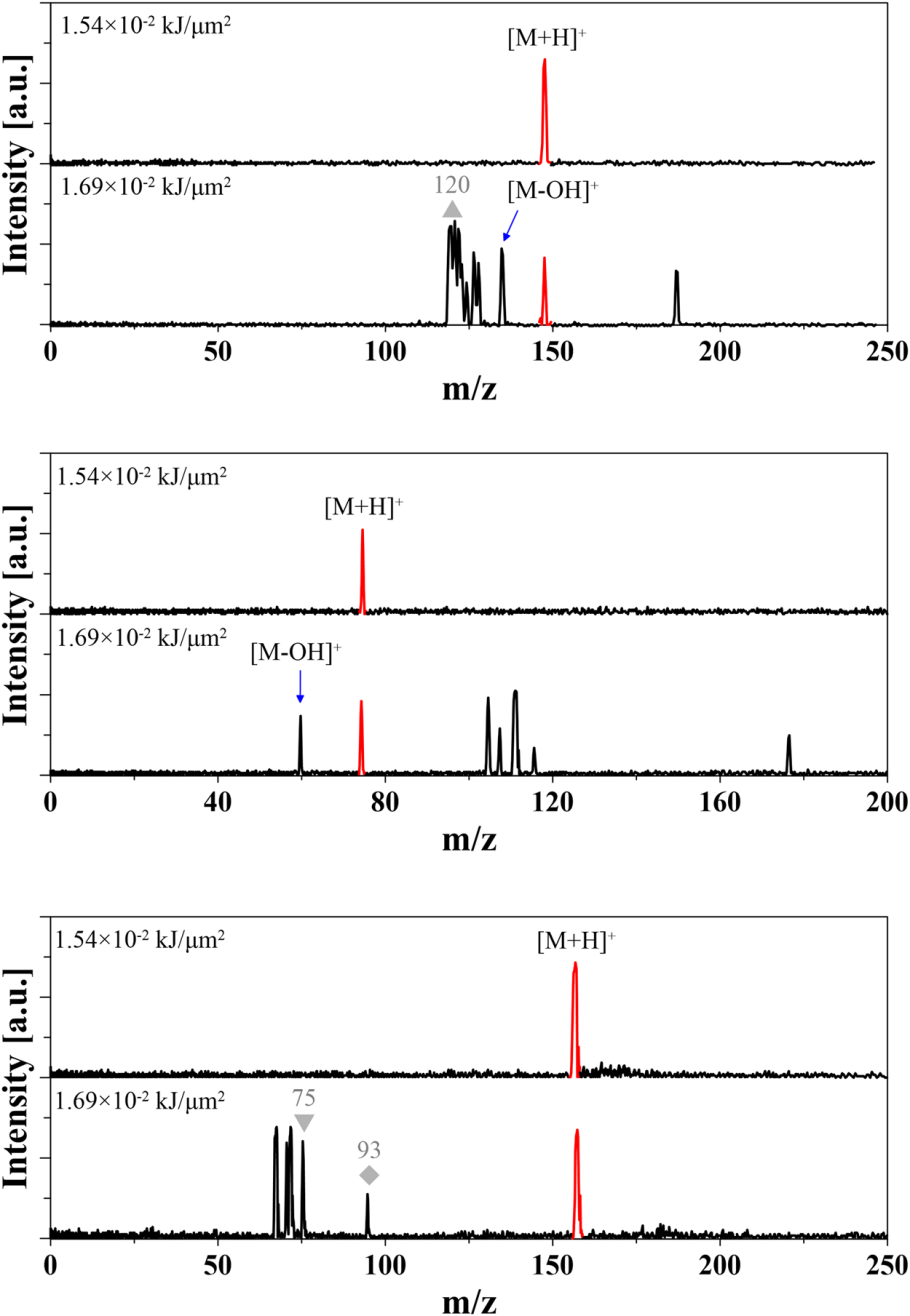
Matrix-free analysis of amino acids by PHDI. (**a**) Glutamic acid; (**b**) Glycine; (**c**) Histidine. Possible fragmentation ion signals are marked with ▲ ([M-H_2_O + H]^+^), ◆ ([C_2_H_4_NO_2_ + H]^+^) and ▼ ([C_2_H_6_NO_3_ + H]^+^).

Matrix-free protein desorption/ionization is aimed at avoiding matrix effects and simplifying the sample preparation process, which is benefit for reliable on-site analysis. Cyt c as a standard protein sample was first tested with matrix-free PHDI method. Figure 2 shows the mass spectra of the Cyt c sample obtained by the PHDI method with different applied pulse-heating energies. No signal was observed at a pulse-heating energy of less than 1.26 × 10^−2^ μJ/μm^2^, although most of the sample in the ionization zone was consumed by the first pulse. Even after a second pulse with higher energy was applied, there was no ion signal, indicating that the sample in the ionization zone was desorbed but without ionization by the first pulse. When a pulse-heating energy of 1.33 × 10^−2^ μJ/μm^2^ was supplied, a nearly ideal mass spectrum with only the singly charged ion signal was obtained. With an increase in the pulse voltage (from 1.33 × 10^−2^ to 1.47 × 10^−2^ μJ/μm^2^), the intensity of the [M ± X]^+^ signal decreased and fragmentation ion signals appeared. This result implies that the generation of ion signals is strongly dependent on the supplied energy. To further confirm the capability for matrix-free protein analysis, BSA, α-lactalbumin (α-Lac, 14.2 kDa) and angiotensin I peptide (1296.5 Da) were examined by the PHDI, as shown in SI-Figs 2, 3 and 4. With an increase in the supplied pulse-heating energy, a similar tendency as that for the Cyt c sample was observed. For instance, only fragmentation ion signals were observed from the BSA sample, although sample desorption and ionization processes occurred. Further optimization of the sample concentration and thickness may be required for obtaining singly charged ion signals. In the case of the Cyt c sample, a higher energy was needed for the matrix-free PHDI method (Fig. 2) as compared with that of the matrix-assisted PHDI method (SI-Fig. 1).

### Ionization of carbohydrates

Carbohydrates play many critical roles in biological processes, such as cell-cell recognition, protein targeting, and nutrition^23^. Developing a method which can analyse carbohydrates becomes increasingly important for understanding their intracellular biological functions. However, such carbohydrates are practically difficult to analyse by MALDI because of two main reasons. One is the strong hydrophilic nature of the carbohydrate and lack of acidic or basic groups to combine with a proton^7,20^. The other reason is the interference caused by the matrix signal in the low mass region. Generally, carbohydrates are derivatised to increase their volatility and stability for MS analysis. However, the derivatization process requires tedious sample purification steps, which may lead to sample loss^24^. In the preliminary test on the capability of our PHDI method for carbohydrates analysis, glucose (1 mg/mL) and α-cyclodextrin (α-CD, 1 mg/mL) as representatives were analysed without derivatization or matrix addition. Figure 3a shows the mass spectra of glucose with two levels of energy supplied. Under the optimized conditions, the singly charged ion signal was obtained with a small degree of fragmentation, illustrating the excellent performance of the PHDI method. Similar to the case of the protein samples, under higher energy, fragmentation ion signals were observed. Figure 3b shows the mass spectra of the α-CD sample with two levels of energy. Compared with the glucose sample, much higher energy was required for desorption and ionization of α-CD. In addition, not only the singly charged α-CD ion signal but also the singly charged glucose unit signal was observed. As the energy increased, fragmentation occurred as a result of cleavage of the glucose units (G)^25^.

### Ionization of amino acids

Amino acids play important roles as building blocks of peptides and proteins. Single amino acids also participate in cell and energy metabolism^26^. Therefore, an effective method for amino acids analysis is important to understand their behaviour and functions in biological systems. In this work, three kinds of amino acids were tested by the matrix-free PHDI method, including glutamic acid (Glu), Glycine (Gly), and Histidine (His). Figure 4 shows the mass spectra of the amino acid samples with different energies supplied. Under the optimized conditions, singly charged ion signals were observed from all samples. When the supplied energy was increased, fragmentation ion signals appeared. For the Glu and Gly samples, the [M-OH]^+^ signal was observed.

## Discussion

**Figure 5:**
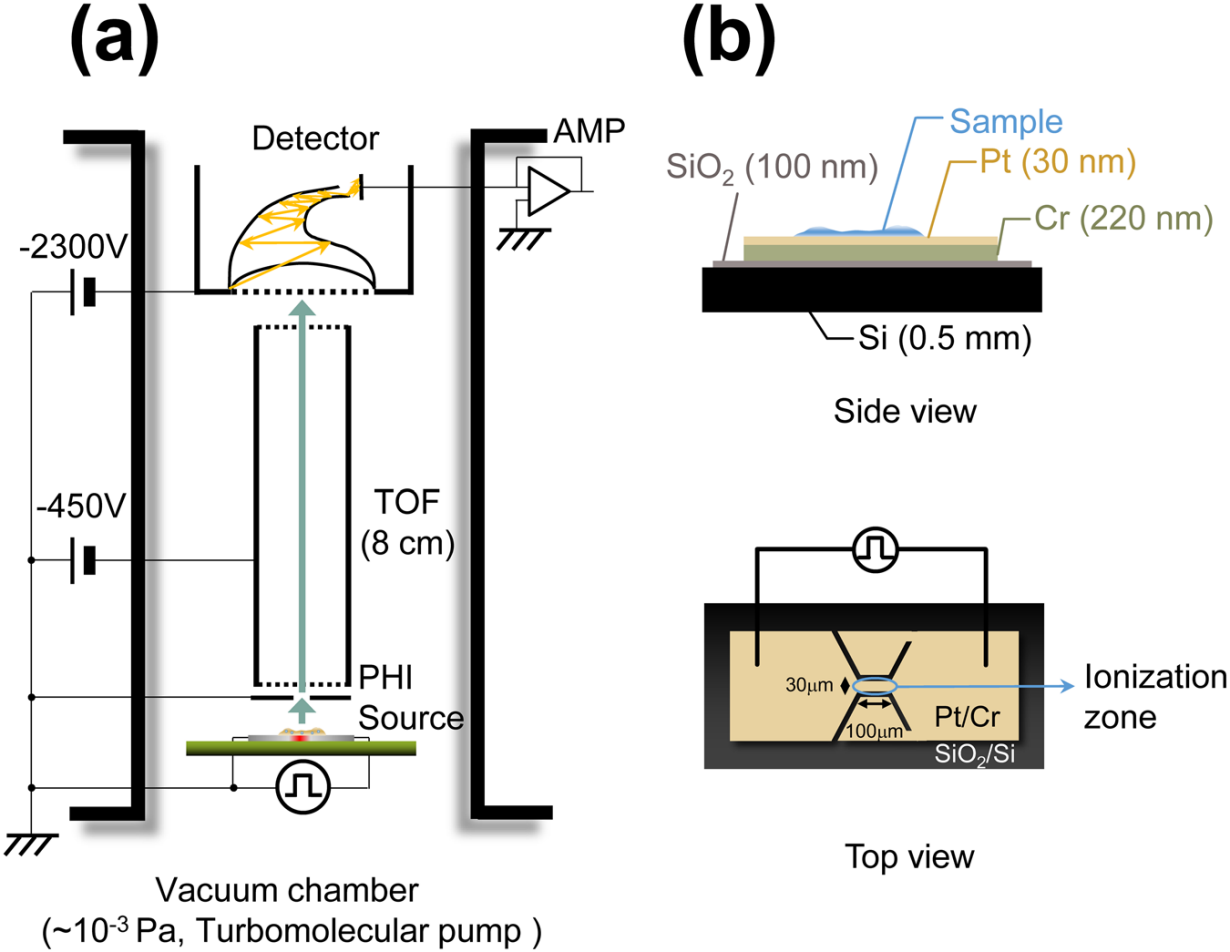
Schematic of on-chip PHDI-MS system configuration. (**a**) Main component of the MS system. (**b**) Side and top views of the on-chip PHDI source.

As compared to a previous study, the present experiments yielded a much clear mass spectrum with very few fragmentation ion signals^19^. One possible reason for the same is that a more homogeneous ion generation process was enabled by the uniform and thin sample film, as opposed to the non-uniform and thick (300 nm) BSA film used in the previous study^19^. In this study, the oxygen plasma treatment of the micro-heater surface was introduced before sample layer formation, so that the water wettability of the PHDI chip could be increased and the sample film thickness was significant reduced to approximately 20 nm. Thus, the thermal energy supplied by the micro-heater could be effectively transferred and uniformly distributed over the thin sample film. This is in contrast to a previous study, where the thermal distribution was less uniform due to the non-uniform and thick film, which could result in fragmentation. In addition, the reproducibility of the mass spectrum was greatly improved by the uniform sample film. A typical sample-to-sample reproducibility of the PHDI is shown in SI-Fig. 5.

Regarding the ionization yield, the PHDI method gave a series of quantitative peaks with a single shot of pulse-heating (Figs 1–4 and SI-Figs 1–4), and clear peaks were observed even with low sample concentrations, which give a small peak in conventional MALDI (Fig. 1-b and SI-Fig. 1-a); thus, the ionization yield with PHDI is not poor. For further discussion, the sensitivity of the PHDI method was estimated from the sample consumption per pulse. Taking the BSA sample as an example, a dried sample spot (~2 mm in diameter) could be formed with 250 nL BSA solution (0.1 mg/mL). For each pulse-heating, the sample in the ionization zone (30 μm × 100 μm) was completely desorbed and ionized. Therefore, approximately 360 amol BSA was consumed per pulse-heating shot, which is comparable with the sample consumption (23–1000 amol) per laser shot in conventional MALDI^27–29^. As shown in Fig. 1, a relatively high S/N was obtained by the PHDI method with only one pulse, as opposed to the MALDI method, which required 10,000 shots. Therefore, the ionization yield for PHDI was considered much higher than that for the MALDI method for each pulse (shot). A possible reason is as follows. In our previous study (shown in Fig. S4-a in the paper ref.^13^), desorption and ionization occurred at a limited range of higher pulse-heating energy (from 1.45 × 10^−2^ to 1.65 × 10^−2^ μJ/μm^2^), and only desorption occurred at a wider and lower range of pulse-heating energy (from 1.02 × 10^−2^ to 1.45 × 10^−2^ μJ/μm^2^)^19^. If a similar phenomenon would occur in MALDI, it is considerable that only the sample at the centre of the laser spot would be desorbed and ionized, while the other part was desorbed only because the laser energy varied strongly in the laser beam profile. On the contrary, the thermal energy for PHDI is uniformly distributed over the ionization zone. Therefore, the PHDI method is more efficient than the MALDI under the optimized conditions.

The capabilities of matrix-free on-chip PHDI paradigm was unfolded successfully for a wide variety of biomolecules, which indicated that the matrix is not essential for the PHDI method. For proteins such as Cyt c, a relatively high thermal energy is required for the matrix-free PHDI (Fig. 2) as compared with the matrix PHDI (SI-Fig. 1). This result suggests that the matrix assists desorption/ionization to some extent and probably donates protons to the protein molecules, similar to the conventional MALDI^30,31^. For BSA (SI-Fig. 3), singly charged ion signal was not observed without matrix. For such large proteins, singly charged ion signals could be obtained with the matrix. Besides, further optimization of the sample concentration and thickness may help obtain singly charged ion signals without matrix. In short, the matrix-free PHDI method is suitable for small molecules, while analysis of large molecules requires a matrix to obtain the singly-charged ion signals.

The supplied thermal energy is an important parameter for the ion generation mechanism in the PHDI method. Desorption occurs at a lower thermal energy, and with an increase in the supplied thermal energy, ionization starts. At the appropriate level of thermal energy, only singly charged ions are generated. At high energy, fragmentation ion signals may be observed due to decomposition and fragmentation. As reported in our previous study^19^, when 1.68 × 10^−2^ μJ/μm^2^ of pulse-heating energy was applied, the temperature of the ionization zone was approximately 2180 K (the melting point of Cr), which is comparable with that in MALDI. According to a report^32^, around 80% of the initially absorbed UV laser energy in MALDI is converted to thermal energy. Recently, a review related to the thermal ionization mechanism of MALDI was published^33^. It introduced that the temperature for primary ion formation in the gas-phase is 1,900 K in the case of DHB. Therefore, the mechanism of the PHDI method is considered to be somewhat similar to the thermal ionization occurring in the MALDI. However, further studies are needed for understanding the mechanism of the PHDI.

In the future, the on-chip PHDI ion source could be combined with other miniaturized components (pump^34,35^, mass filter^36–38^, detector^39,40^, etc.) to realize a one-chip MS system. Besides, the capability to observe singly charged ions and fragmentation ions by thermal energy alone opens a new perspective on the ionization mechanisms for conventional MALDI. The simple and effective ionization method is strongly expected to trigger the next stage of development of MS technology.

In conclusion, this study has demonstrated the feasibility of using the PHDI method for matrix-free desorption/ionization of a wide variety of biomolecules, including carbohydrates, which are difficult to analyse directly by the conventional MALDI. With a uniform and thin sample film, the on-chip PHDI method shows high yield and affords singly charged ions with very less fragmentation and background with one pulse-heating shot. Without the matrix, the sample preparation could be simplified for the on-chip PHDI source, making it suitable for application to on-site and rapid analysis.

## Methods

### On-chip PHDI ion source MS system

The on-chip PHDI source consisting of a Pt/Cr micro metal electrode layer was fabricated by a lift-off processing on a SiO_2_/Si substrate^19^. A narrow part (length: 100 μm, width: 30 μm) of the Pt/Cr electrode layer acted as the ionization zone. In previous study, a SiO_2_ insulating layer covering the ionization zone was used to form a uniform hydrophilic surface. However, the insulating layer may affect the efficiency and uniformity of thermal conduction. Alternatively, oxygen plasma treatment was introduced for cleaning the surface and increasing the water wettability of the ion source chip before sample film formation^41^. After each experiment, the chip was carefully cleaned by sonication in ethanol and pure water for 3 min, for reuse.

The main components of the miniaturized mass spectrometer, including the on-chip ion source, linear time-of-flight (TOF) mass filter (8 cm) and a Channeltron electron multiplier (CEM) as the detector (Detector Technology, Model 414, USA), were placed in a vacuum chamber (~10^−3^ Pa)^19^. A single pulse voltage (20–45 V, width: 500 ns) was applied to the on-chip ion source for ionization by a function generator (AFG3251, Tektronix Inc., USA). The generated ions were extracted and accelerated by using the potential difference between the surface of the chip and the entrance of the TOF. The TOF signals detected by the CEM were amplified by an amplifier unit (C9663, Hamamatsu photonics, Japan), and then recorded by an oscilloscope (TDS2022C, Tektronix Inc., USA). The mass spectra were calculated from the TOF signals, as previously reported^19^. The acceleration voltage used for all experiments was 450 V. A chip holder was used to fix the chip position for improving the accuracy of the alignment between the ionization source and the mass analyser, so that signal misalignment could be reduced. All mass spectra were obtained by applying only one low-voltage pulse for ionization. Figure 5 shows the schematic of the miniaturized MS system.

### Sample preparation

Unless specified, the samples were prepared according to the following method. 2, 5-Dihydroxybenzoic acid (DHB), sinapic acid (SA), and 2, 5-dihydroxyacetophenone (DHAP), which were used as the matrix, were obtained from Wako Pure Chemical and dissolved in acetone at a concentration of 10 mg/mL. Cytochrome C (Cyt c), α-lactalbumin (α-Lac), and bovine serum albumin (BSA) as the protein samples were prepared by dissolving in pure water at concentrations of 1.0 mg/mL, 1.0 mg/mL and 0.1 mg/mL, respectively. Then, the protein and matrix sample solutions were directly mixed in a ratio of 1:1 (v/v). Glutamic acid (99%), glycine (99%), and histidine (98%) as the amino acid samples were dissolved in pure water at a concentration of 1 mg/mL. Glucose (anhydrous, 98%) and α-cyclodextrin (97%) as the carbohydrate samples were dissolved in pure water at a concentration of 1 mg/mL. Angiotensin I (97%) as the peptide sample was dissolved in pure water at a concentration of 1 mg/mL. After oxygen plasma treatment of the on-chip PHDI source, 250 nL of the sample solution was dropped on the centre (ionization zone) of each on-chip ion source and dried in a vacuum. Thus, a uniform and thin sample film with a thickness of approximately 20 nm was obtained.

### Data availability

The datasets generated and/or analysed in the current study are available from the corresponding author on request.

## Electronic supplementary material

Supplementary Information
