# Supplementary material for: High yield matrix-free ionization of biomolecules by pulse-heating ion source
Source: Sci Rep. 2017 Nov 9;7:15170. doi: 10.1038/s41598-017-15259-y (PMC5680173; doi:10.1038/s41598-017-15259-y)
Supplement: Supplementary file 1 — Supplementary Information [file 41598_2017_15259_MOESM1_ESM.pdf]

Supplementary Information of

High yield matrix-free ionization of biomolecules by pulse-heating ion source

Xi Luo, Phan Trong Tue, Kiyotaka Sugiyama, Yuzuru Takamura*

School of Materials Science, Japan Advanced Institute of Science and Technology (JAIST), 1-1 Asahidai, Nomi, Ishikawa 923-1211, Japan

*Corresponding author.

Tel.: +81-761-51-1661; Fax: +81-761-51-1665.

E-mail address: takamura@jaist.ac.jp

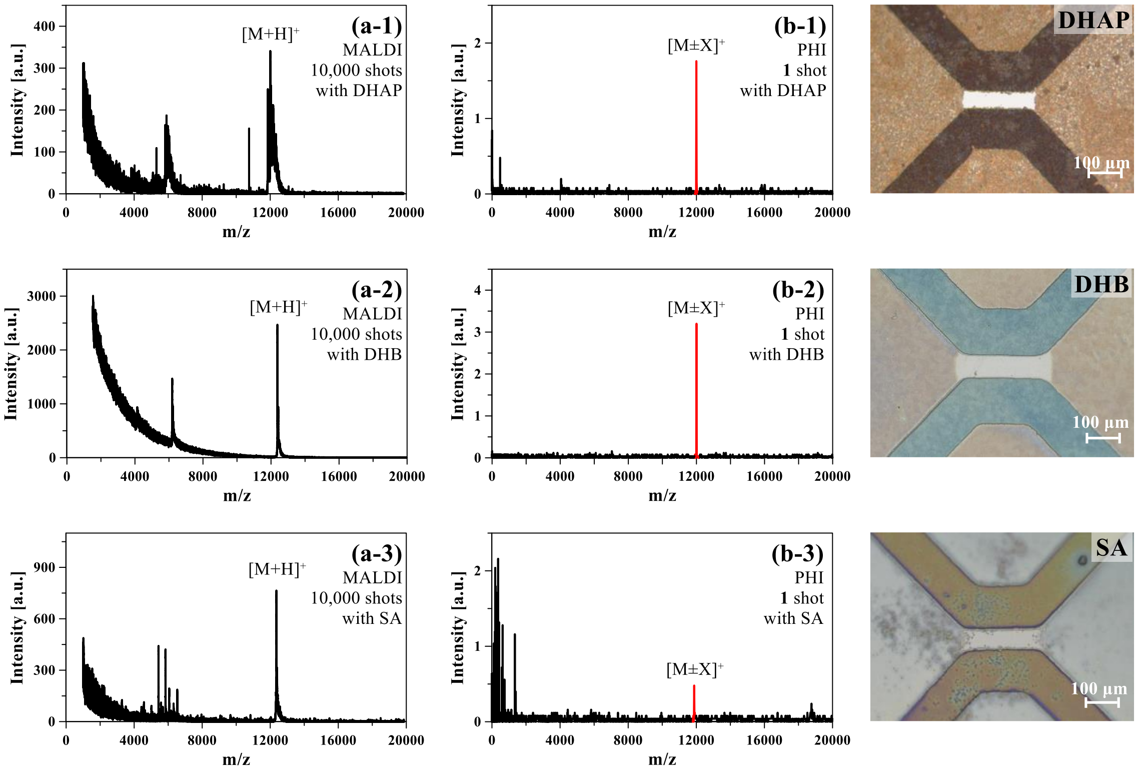

SI-Figure 1. Comparison of conventional MALDI and PHI method. (a) MALDI mass spectra obtained from Cytochrome C (Cyt c) (1.0 mg/mL) mixed with DHAP (a-1), DHB (a-2), and SA (a-3) by 10,000 shots averages. (b) PHI mass spectra obtained by Cyt c (1.0 mg/mL) mixed with DHAP (b-1), DHB (b-2), and SA (b-3) by 1 shot. Inset figures show the microscopic image of PHI source. After one pulse-heating shot, the sample at the centre (ionization zone) was desorbed and ionized.

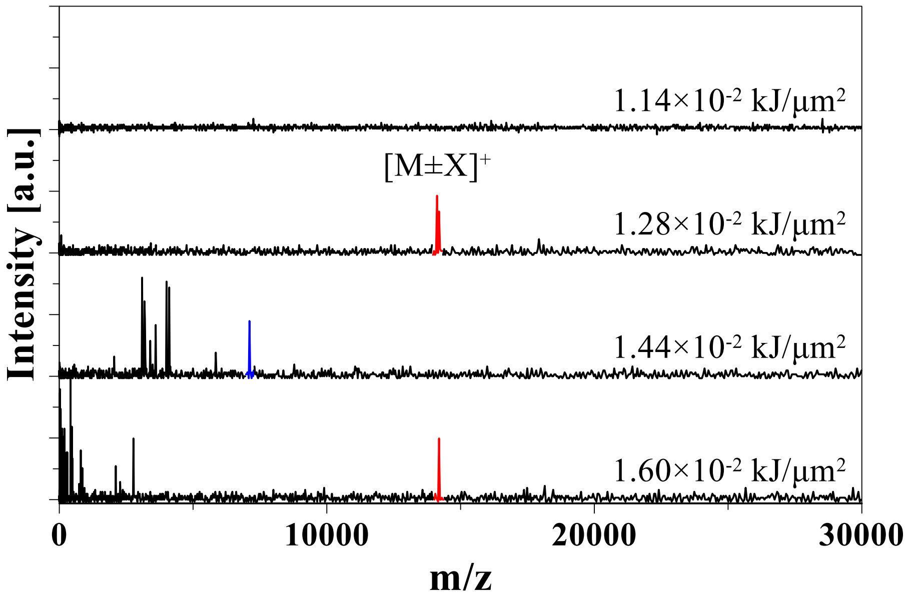

SI-Figure 2. Matrix-free α-Lac analysis by PHI with different levels of pulse-heating energy.

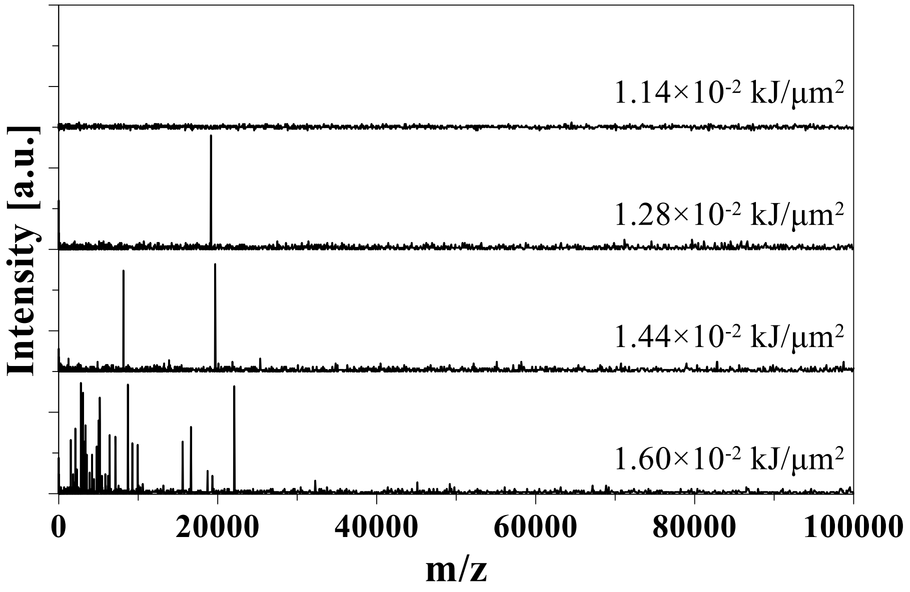

SI-Figure 3. Matrix-free BSA analysis by PHI with different level of pulse-heating energy.

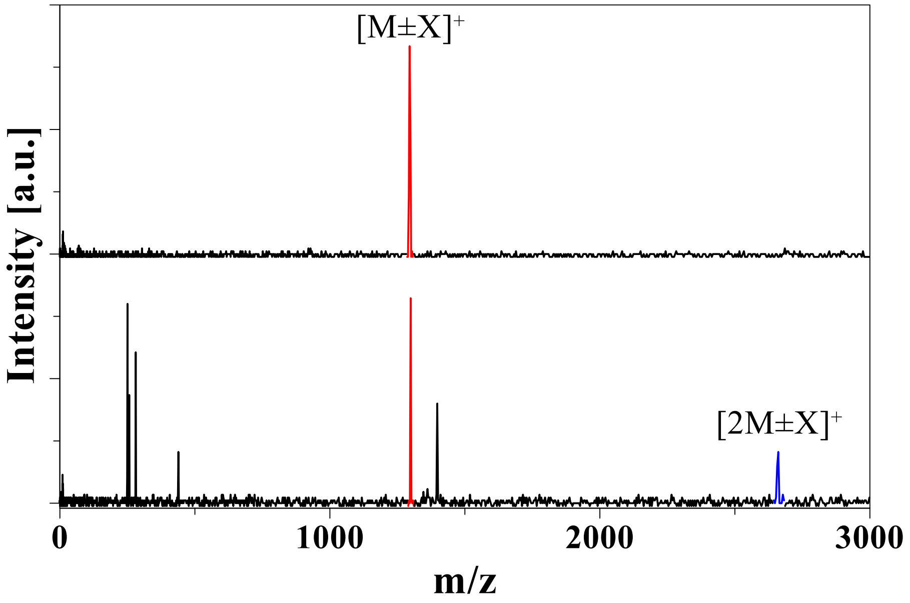

SI-Figure 4. Matrix-free angiotensin I analysis by PHI with different level pulse-heating energy.

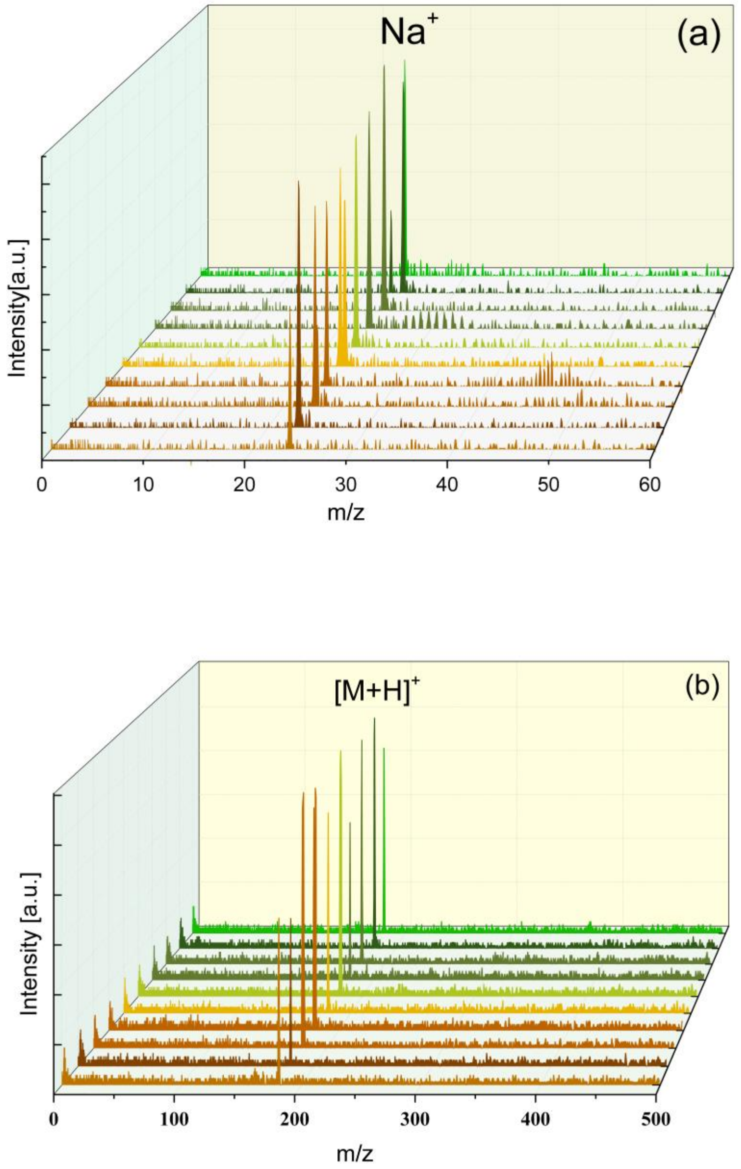

SI-Figure 5. A typical sample-to-sample reproducibility mass spectra of the PHI in positive ion detection mode. (a) Sodium chloride. (b) Glucose.
